# Electrophysiological correlates of mindfulness in patients with major depressive disorder

**DOI:** 10.3389/fnins.2022.971958

**Published:** 2022-10-13

**Authors:** Jan Sarlon, Annette B. Brühl, Undine E. Lang, Andreas Kordon

**Affiliations:** ^1^Department of Adult Psychiatry, University of Basel, University Psychiatric Clinics (UPK), Basel, Switzerland; ^2^Oberbergklinik Hornberg, Hornberg, Germany

**Keywords:** mindfulness, emotional stress, autonomous reactivity, depressive disorder, electrophysiological parameters

## Abstract

**Objectives:**

Mindfulness-based interventions (MBI) can reduce both stress and depressive symptoms. However, the impact of mindfulness on stress level in depressed subjects remains unclear. This study aims to assess electrophysiological correlates of mindfulness in patients with major depressive disorder (MDD) at baseline, under stress exposure, and in relaxation following stress exposure.

**Methods:**

Perceived mindfulness was assessed with the Freiburger Mindfulness Inventory (FMI) in 89 inpatients (mean age 51) with MDD [mean Beck Depression Inventory (BDI) 30]. Electrophysiological parameters [resting heart rate (RHR), heart rate variability (HRV), respiration rate, skin conductance, and skin temperature] were recorded at 5-min baseline, 1-min stress exposure, and 5-min self-induced relaxation.

**Results:**

Freiburger Mindfulness Inventory was strongly inversely correlated with symptom severity measured by BDI (*r* = –0.53, *p* < 0.001). No correlations between FM score and electrophysiological parameters in any of the three conditions (baseline, stress exposure, relaxed state) could be found. The factor openness was associated with higher VLF (very low frequency of HRV) in the baseline condition. However, this correlation was no more significant after regression analysis when corrected for respiratory rate, age, and sex.

**Conclusion:**

Autonomous nervous reactivity in depression was not associated with perceived mindfulness as measured by FMI score and presented electrophysiological parameters, despite the strong inverse correlation between state mindfulness and symptom severity.

## Introduction

Mindfulness-based interventions (MBI) integrated the essence of traditional mindfulness practice with contemporary psychological practice and have recently gained much interest in the scientific world and in the therapy of depression (Hofmann et al., 2010; Gu et al., 2015; Segal and Walsh, 2016; Bulzacka et al., 2018; Cladder-Micus et al., 2018; Geurts et al., 2020; Ninomiya et al., 2020).

The mindfulness literature to date shows considerable variance in how mindfulness can be defined. Mindfulness may be conceptualized as a state, a trait, or an outcome of an intervention (Scavone et al., 2020). Additionally, the dimensionality of mindfulness is debated, with some researchers emphasizing one-dimensionality (Brown and Ryan, 2003) and others assuming multidimensionality (Baer et al., 2006). Mindfulness is usually defined by two core dimensions—present-moment attention and non-judgmental acceptance (Kabat-Zinn, 1994; Ludwig and Kabat-Zinn, 2008; Chambers et al., 2009; Davidson, 2010). An often cited definition of mindfulness is “paying attention in a particular way: on purpose, in the present moment, and non-judgmentally (Kabat-Zinn, 1994).

In literature, many possible hypothesized mechanisms underlying the effects of MBI in depression can be found: decrease in repetitive negative thinking (Gu et al., 2015), emotional regulation *via* exposure and extinction/habituation (Hölzel et al., 2011; Treanor, 2011), decentering (Fresco et al., 2007), self-kindness, and self-compassion (Neff, 2003; Hölzel et al., 2011; Van Dam et al., 2011) as well as a wider range of coping skills (Shapiro et al., 2006; Vago and Silbersweig, 2012).

### Physiological changes and mindfulness

There is evidence that mindfulness interventions are associated with reduced stress levels (Tang et al., 2009; Arredondo et al., 2017; Pascoe et al., 2017; Lengacher et al., 2019; Krick and Felfe, 2020). The outcomes included cortisol, inflammatory as well as electrophysiological parameters.

The previous studies assessing stress *via* electrophysiological parameters, use mostly either heart rate variability (HRV) (Watford et al., 2020; Bortolla et al., 2021; Park et al., 2021) or skin conductance (SC) response (Kadziolka et al., 2016). Both parameters dominate the arousal assessment in depression and suicidality: Recent review by McCall et al. (2022) regarding ANS assays and suicidality included 21 studies, 14 of them used HRV, and six studies used skin conductance as a biomarker. In MBI research, HRV has become an increasingly utilized biomarker (Christodoulou et al., 2020).

HRV is a measure of the variability in intervals between consecutive heartbeats is an indirect assessment of cardiac autonomic activity (Sztajzel, 2004; Billman, 2011; Koenig and Thayer, 2016). Similarly, impaired/lower HRV has been repeatedly associated with stress, depression as well as other psychiatric conditions (Lin et al., 2011; Alvares et al., 2016; Hartmann et al., 2018; Huang et al., 2018; Järvelin-Pasanen et al., 2018; Heiss et al., 2021). Most of he research in this field uses time and frequency domains, alternative measures include geometric and non-linear measures (Heart Rate Variability, 1996; Kleiger et al., 2005). It must be emphasized that HRV only provides an indirect assessment of cardiac autonomic activity and does not provide a direct measurement of either cardiac parasympathetic or sympathetic activity (Billman, 2013). Studies with non-clinical samples that included HRV assessment demonstrated mostly positive effects of MBI on HRV immediately after the intervention (Christodoulou et al., 2020; Krick and Felfe, 2020) as well as in follow-up at week 20 (Arredondo et al., 2017). Similarly, an increase in HRV after Zen or Vipassana meditation compared to baseline has been reported (Wu and Lo, 2008; Krygier et al., 2013).

However, there are also contrary findings (Nijjar et al., 2014), partly also due to difficulties comparing different conditions and methods (Voss et al., 2020). A meta-analysis of the effects of standardized MBI by focusing on inflammatory parameters and HRV revealed mixed and inconclusive results (Rådmark et al., 2019). Some authors demonstrated robust effects of MBI on stress when measured *via* self-report, but less clear for physiological measures (Morton et al., 2020). Despite the rising popularity of HRV measurements in the assessment of stress, there are rising concerns regarding high levels of within-subjects variability in HRV measurements (Uhlig et al., 2020), a variety of HRV measurement protocols (Vila et al., 2019), as well as poorer reliability in real-world recordings (von Rosenberg et al., 2017) and in clinical populations (Sandercock et al., 2005) compared to lab-based scenarios or healthy populations. Some authors suggest the use of broader analyzing tools instead of HRV only (Schumann et al., 2017; Sarlon et al., 2018).

### Mindfulness in non-clinical and clinical samples

The vast majority of studies addressing the link between mindfulness and physiological changes are based on healthy populations (Watford et al., 2020; Bortolla et al., 2021; Park et al., 2021). If a stress induction task is used, participants are also mostly volunteers (Paul et al., 2013; Feldman et al., 2016; Sun et al., 2019). A widely spread approach is also to compare meditation/mindfulness interventions with the control condition (Magalhaes et al., 2018; Belliveau et al., 2021; Cohen et al., 2021; Saltsman et al., 2021) or comparing experienced meditators with novices (Lutz et al., 2016; Rodriguez-Larios et al., 2021).

In clinical samples (patients with relevant depression), mindfulness-based cognitive therapy reduced depressive symptoms but did not let to significant changes in HRV (Wheeler). A recent study on depressed patients of Belliveau et al. (2021) showed that mindfulness-based cognitive therapy in depressed may not be accompanied by changes in the stress-response and inflammatory pathways.

As mentioned above and with regard to the current research of mindfulness and stress/depression, the following main concerns can be expressed, indicating a potential knowledge gap:

• Most of the findings in the literature concern healthy subjects and less depressed patients.
• Findings in clinical samples exploring the effects of mindfulness on HRV are partly contrary to healthy subjects but also in non-clinical samples are the results inconclusive.
• The design of the most studies assessing the influence of mindfulness is comparative/interventional; there is a lack of empirical studies examining state mindfulness.
• Even if popular in German-speaking countries, the Freiburger Mindfulness Inventory (FMI) is usually not a part of the studies assessing mindfulness.

The aim of the present study is, therefore, to assess electrophysiological correlates of perceived mindfulness in patients with major depressive disorder (MDD) using static as well as dynamic measures (indices of the autonomous nervous reactivity) and FMI.

FMI was initially introduced as a 30-item, in the short version a 14-item instrument assessment of present moment attention, non-judgment, and openness (Baer et al., 2006; Walach et al., 2006) and is widely spread in German-speaking clinical settings.

Although our study was primarily explorative, there was one specific hypothesis we aimed to prove. Assuming a potential mediating role of autonomous nervous system activation, we specifically hypothesized that the global mindfulness score (as assessed by FMI) would have an impact on the measured stress level in all three conditions, especially under stress exposure.

## Materials and methods

### Transparency and openness

In the following section, we report how we selected our sample, all data exclusions, all manipulations, and all measures in the study (Simmons et al., 2011). The data used to derive our statistical inferences are provided as Supplementary material. The study’s design and its analysis were not preregistered.

### Participants

Over the duration of the study, all new inpatients with MDD were asked to participate. Altogether, 98 inpatients with a diagnosis of unipolar MDD, according to the International Classification of Diseases (ICD-10, 10th edition), based on the clinical examination and interview, entered the study. Exclusion criteria were: bipolar disorder (one subject excluded), other manifest psychiatric disorder as the main diagnosis (three subjects excluded), manifest diabetes mellitus (one subject excluded), manifest neurological disorder (multiple sclerosis, one subject excluded), pathological thyroid-stimulating hormone blood level (two subjects excluded), elevated leukocyte counts (one subject excluded) as well as not compensated arterial hypertonia or heart disease, cancer or other serious medical condition. In case of alcohol use in the history, abstinence from alcohol in the past 4 weeks was required.

The final sample consisted of 89 inpatients (37 women = 42%, mean age 51 years, *SD* = 11.28, range 23–69). Comorbidity of arterial hypertension was observed in 15 patients, 9 of them on medication. Seven subjects had a history of hypothyroidism, six of them were substituted (levothyroxine). Lumbar syndrome (previous disc prolapse) was reported in three cases, allergy or asthma in six subjects. A total of 31 patients were drug naive, and 58 patients were on psychiatric medication. Antidepressants taken were selective serotonin reuptake inhibitors (SSRI) [escitalopram (12), sertraline (7), citalopram (3)], venlafaxine (13), mirtazapine (13), bupropion (6), vortioxetine (2), and tianeptine (2). Other medications consisted of antihypertensive drugs (nine patients), levothyroxine (six patients), proton pump inhibitors (three patients), asthma spray (in two patients), acetylsalicylic acid, and diclofenac each in one patient. All patients received treatment as usual (TAU) including progressive muscular relaxation or short relaxation 60–90 min per week and have been introduced to the theory of mindfulness in educative group sessions. Specific mindfulness-based therapy (like mindfulness-based stress reduction, MBSR or mindfulness-based cognitive therapy, MBCT) did not take place. Although PMR was a part of the TAU, no slow breathing has been taught. The previous experience with MBI was not systematically assessed.

### Study design

Overall, we used a repeated measurement design including three time points of measurement: baseline, during stress exposure, and during a relaxation state.

In our study, we decided to use a recall of unpleasant stressful experiences of a medium intensity as a *stress exposure*. We asked participants to imagine an unpleasant stressful situation from the past of medium intensity and to mark the stress intensity on a visual analog scale (VAS, a horizontal line, 100 mm in length, anchored by word descriptors at each end, and numbered from 0 to 10. The endpoints were “not stressful at all” and “extremely stressful”). The VAS score was determined by measuring in cm from the left end of the line to the patient’s mark. The mean stress intensity measured by VAS was 4.85 (*SD* = 0.67).

A relaxed state followed the stress exposure: patients should try to relax as much as possible, either with their eyes closed or open, staying in the same position and avoiding all unnecessary movements. They were asked to use their usual strategies to relax as they do in their daily life or in the hospital.

### Measurement protocol

To assess the severity of depressive symptoms, the German version of the BDI (Kühner et al., 2007) was used. Mindfulness was measured using the FMI. Both self-rating tests were administrated prior to the electrophysiological measurements.

Skin conductance (SC) and skin temperature (ST), resting heart rate (RHR), HRV as well as respiration rate (RR) were recorded for 300 s baseline, 60 s stress exposure, and 300 s self-induced relaxation.

### Measurements

Self-reported data (i.e., depression symptoms and mindfulness) were assessed prior to the electrophysiological measurements at baseline. Electrophysiological measurements were recorded as described above.

### Self-reported data

#### Beck Depression Inventory II

Beck Depression Inventory II is a revised version of the depression inventory developed by Aaron Beck and consists of 21 items. BDI-II items are rated on a 4-point scale ranging from 0 to 3 based on the severity of each item. The maximum total score is 63. The internal consistency was described as around 0.9 and the retest reliability ranged from 0.73 to 0.96 (Wang and Gorenstein, 2013).

#### Freiburger Mindfulness Inventory

We used the shortened form with 14 items with a Cronbach alpha of 0.86 (the initially 30-item scale with an internal consistency of Cronbach alpha = 0.93) (Walach et al., 2006). Participants should provide an answer for every statement as best they can, choosing one of the following answers: Rarely—occasionally—fairly often—almost always. The score for each item varies from one to four points, given a range of total scores between 14 and 56.

### Electrophysiological data

Electrophysiological data were acquired using the NeXus-10 system (NeXus-10 Mark II^®^, Biotrace +) and were recorded at a sampling rate of 1,024 Hz. For heart rate and HRV recording, a blood volume pulse finger clip sensor was used. To measure SC, Velcro tape with integrated Ag/AgCl electrodes was placed at the middle phalanx of the index finger and the ring finger of the left hand. For the measurement of ST, the NeXus temperature sensor was placed on the middle finger of the left hand and taped to the finger at two positions to ensure stability.

To measure RR, an elastic belt with a breathing sensor was fixed around the lower thorax at the diaphragm, according to the manufacturer’s instructions. The same procedures were used for all study participants to ensure that the data from all individuals were comparable. All measurements were taken in the same room and under the same conditions: sitting, slightly inclined position with eyes open. During the baseline recording, all subjects were asked to breathe normally, not talk, and to avoid all unnecessary movements. Furthermore, to reduce the impact of pre-test activities, they were asked to sit and not move for 5 min prior to placement of the electrodes.

### Pre-processing and analysis

Recording and primary analysis of all electrophysiological parameters were performed using the BioTrace + software. For the analysis of HRV, time- and frequency-domain parameters and records 5 min in length with a sampling rate of 1,024 Hz were used, according to recognized standards (Heart Rate Variability, 1996). The power frequency spectrum of HRV was subsequently quantified in standard frequency-domain measurements, such as total variance, high-frequency (HF, 0.15–0.4 Hz), low-frequency (LF, 0.04–0.15 Hz), very-low-frequency (VLF, 0–0.04 Hz), and LF/HF ratio. For the time domain, principal parameters such as beat-to-beat interval (RR or NN-interval), the standard deviation of the beat-to-beat interval (SDNN), and the root mean square of successive differences (RMSSD) were used.

Prior to the analyses, all parameters were visually controlled on a 15-s window, and artifacts in the RHR and HRV parameters due to the movement were removed. Artifacts due to device failure in the SC or ST were also removed. Measurements with more than 5% artifacts were excluded; this was the case in four measurements of SC as well as in five measurements of RHR and HRV parameters.

Statistical analyses were performed using R-Studio software (version 1.2). First, a descriptive analysis has been performed to summarize the sample. Median and mean have been used as indicators of central tendency, range, and standard deviation as indicators of variability. Second, a correlation analysis has been conducted. To choose the appropriate statistical tool, the normality of the distribution was assessed using the Shapiro–Wilk test. Variables have been considered as not normally distributed, it the *p*-value was less than α = 0.05. RR and RHR met the normality assumption. FMI score missed with *p* = 0.047 the criteria, the single items were not normally distributed either. ST, SC, and HRV parameters were also found not to be normally distributed; moreover, they were extremely skewed. For that reason, we log-transformed all electrophysiological parameters, and the log-transformed HRV values were normally distributed. For normally distributed variables, a Pearson correlation analysis was used; non-normally distributed variables were assessed for correlation using Spearman’s correlation. Third, multistep linear regression controlling for age and sex has been performed, using the command “lm” in R-studio (free version 3.6.3 with R stats and *utils, datasets, formal methods, and classes* packages as well as *dplyr* and *ggpubr*).

## Results

**FIGURE 1:**
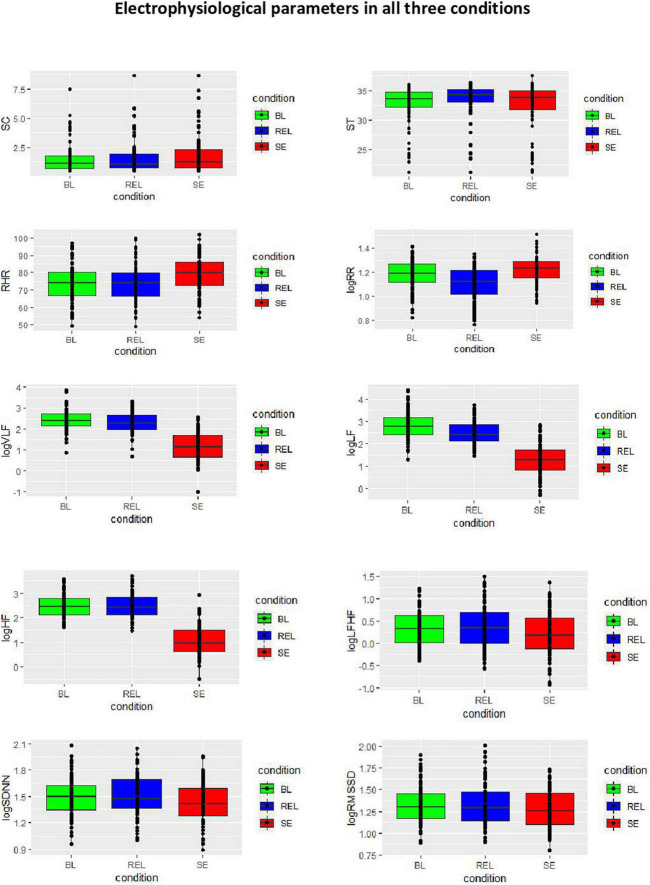
Electrophysiological parameters in all three conditions. SC, skin conductance; ST, skin temperature; RHR, resting heart rate; RR, respiration rate per minute; HRV, heart rate variability; SDDN, standard deviation of beat-to-beat intervals; RMSSD, root mean square of successive differences; VLF, very low frequency; LF, low frequency; HF, high frequency.

The mean FMI score was 32.79 (*SD* = 6.31), the mean symptom severity measured by the BDI-II score was 29.66 (severe depression). Table 1 is summarizing the study population and all electrophysiological parameters, Figure 1 is visualizing the group differences. Significant between-group differences could be found in RHR, RR, SDNN, LF, and HF. For more details, see also our previous work (Sarlon et al., 2021).

**TABLE 1:** Basic characteristics of the study population.

Population	FMI score	BDI score	BMI	Age	Electrophysiological parameters	SC Mean *SD*	ST Mean *SD*	RHR Mean *SD*	RR Mean *SD*	SDNN Mean *SD*	RMSSD Mean *SD*	VLF Mean *SD*	LF Mean *SD*	HF Mean *SD*	LF/HF Mean *SD*
*Mean* *SD*	**32.79** 6.31	**29.66** 10.71	**25.52** 4.02	**50.61** 10.34	(B) Baseline	**1.51** 1.28	**32.54** 3.56	**74.11** 10.62	**15.49** 4.15	**35.58** 19.99	**25.07** 14.68	**543.78** 1,053	**1894.97** 4,052	**624.32** 892	**3.22** 3.23
	**Sex** *F* = 37 (42%)	*M* = 52 (58%)			(S) Stress exposure	**1.79** 1.63	**32.26** 4.16	**79.22** 10.25	**17.05** 4.36	**30.60** 17.00	**21.43** 10.77	* *	**72.90** 143	**38.22** 101	**2.94** 3.61
					(R) Relaxed state	**1.64** 1.50	**33.14** 3.34	**73.80** 10.71	**13.21** 4.11	**38.18** 22.06	**26.19** 18.59	**384.76** 460	**2087.26** 3,393	**602.95** 905	**4.49** 6.02

FMI, Freiburger Mindfulness Inventory (score range: 14–56); BDI, Beck Depression Inventory (score range: 0–63); BMI, body mass index; SD, standard deviation; SC, skin conductance (mS); ST, skin temperature (Grad Celsius); RHR, resting heart rate (per minute); RR, respiration rate (per minute); HRV, heart rate variability; SDDN, standard deviation of beat-to-beat intervals (ms); RMSSD, root mean square of successive differences (ms); VLF, very low frequency (ms^2^); LF, low frequency (ms^2^); HF, high frequency (ms^2^).

*VLF under stress exposure was omitted since this phase was 60 s long and VLF range extends from 25 to 300 s.

Bold represents the mean (under it the SD).

### Correlation analysis of Freiburger Mindfulness Inventory and electrophysiological parameters

The performed correlation analysis (see also section “Materials and methods”) can be characterized by two main steps. At the first step, we correlated the FMI total score with all measured parameters in every condition (baseline, stress exposure, and relaxed state) as well as between the FMI total score and the differences (change scores) of electrophysiological parameters between all three conditions (example: FMI score and the difference between RR at baseline and under stress exposure) (Table 2). A strong, inverse correlation between symptom severity (BDI score) and perceived mindfulness (FMI score) could be observed (Pearson’s *r* = –0.53, *p* = 6.7 × 10^–8^). There was no correlation between FMI score and electrophysiological parameters or other assessed influencing factors.

**TABLE 2:** Correlation of FMI and electrophysiological parameters across the three different conditions.

		SC	ST	RHR	RR	SDNN	RMSSD	VLF	LF	HF	LF/HF
Baseline	R	–0.035	–0.060	–0.017	–0.151	–0.071	–0.071	–0.094	0.00	–0.093	0.212
	Df	83	86	82	86	81	81	81	81	81	81
	P	0.750	0.579	0.88	0.160	0.526	0.526	0.399	0.995	0.401	0.054
Stress exposure	R	–0.034	0.071	0.01	–0.066	0.114	0.106	–0.035	0.045	0.053	0.049
	Df	86	86	82	87	82	82	82	82	82	82
	P	0.751	0.51	0.950	0.540	0.302	0.335	0.754	0.686	0.630	0.655
Relaxed state	R	–0.065	0.026	–0.042	–0.066	0.075	–0.083	–0.175	–0.032	–0.116	0.072
	Df	82	86	80	85	79	79	79	79	79	79
	P	0.556	0.809	0.705	0.543	0.507	0.462	0.119	0.780	0.301	0.521
B—S	R	–0.055	–0.156	–0.003	–0.055	–0.104	–0.126	–0.012	0.119	0.119	0.122
	Df	83	85	79	85	87	87	87	87	87	82
	P	0.601	0.149	0.977	0.616	0.330	0.238	0.909	0.266	0.266	0.270
S—R	R	0.083	0.001	0.022	0.043	0.120	0.121	0.145	0.006	0.001	–0.030
	Df	82	83	79	86	85	85	85	85	85	87
	P	0.442	0.991	0.844	0.694	0.269	0.263	0.181	0.953	0.955	0.781
B—R	R	0.126	–0.137	0.081	–0.057	0.061	0.043	0.054	0.161	0.161	0.057
	Df	82	84	80	87	83	83	83	83	83	82
	P	0.243	0.206	0.462	0.598	0.579	0.699	0.621	0.141	0.1411	0.605

B, Baseline; S, Stress exposure; R, Relaxed state; SC, skin conductance; ST, skin temperature; RHR, resting heart rate; RR, respiration rate; SDDN, standard deviation of beat-to-beat intervals; RMSSD, root mean square of successive differences; VLF, very low frequency of heart rate variability (HRV); LF, low frequency of HRV; HF, high frequency of HRV. R, correlation coefficient; Df, degree of freedom; *P*, *p*-value.

*R* < 0.100 or > –0.100 no fill. 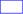

*R* < 0.200 or > –0.200 light orange fill. 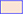

*R* > 0.200 or < –0.200 orange fill. 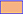

**FIGURE 2:**
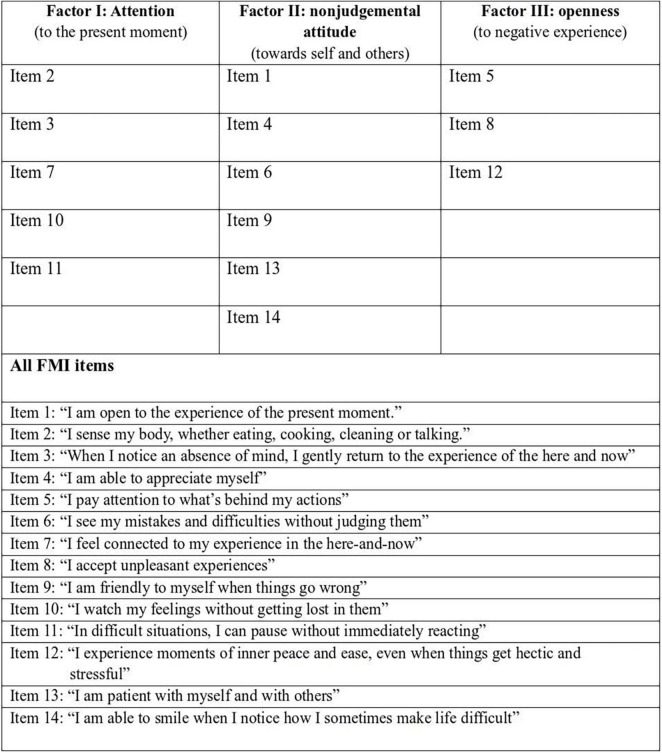
Items and factors of FMI (Freiburger Mindfulness Inventory).

In the next step, we compared the three main factors as postulated by the authors of FMI (see Figure 2) with the electrophysiological parameters in different stages. The third factor, “Openness” correlated with VLF at baseline (Spearman’s rho = –0.246, *p* = 0.025), no other correlation could be observed.

Assessed HRV indicators are described above (see section “Pre-processing and analysis”). In the following, some HRV indicators are briefly summarized. Both parasympathetic and sympathetic systems are believed to contribute to SDNN, which is highly correlated with ULF, VLF, and LF. For short-term recording, ULF will not be assessed. The RMSSD is believed to be more influenced by parasympathetic system than sympathetic system than SDNN. Experimental evidence suggests that VLF is modulated by physical activity and stress responses. The HF band reflects parasympathetic activity and corresponds to the HR variations related to the respiratory cycle (Shaffer and Ginsberg, 2017). The inclusion of LF/HF-ratio in our overview has a historic background (previously suggested as an index of sympatho–vagal balance). At this point it should be clearly stated, that available data challenge this interpretation, showing that LF is determined by both sympathetic and parasympathetic systems as being an index of sympathetic activity (Houle and Billman, 1999; Heathers, 2012; Billman, 2013; Reyes del Paso et al., 2013).”

### Influencing factors and electrophysiological parameters

The role of influencing factors was an object of the previous author’s work (Sarlon et al., 2021).

Briefly summarized, symptom severity measured by BDI score was associated with higher SC under stress exposure and with a lower RR in the relaxed state. Age and higher blood pressure were both associated with lower HRV and higher RHR. Significant gender differences could be demonstrated in the relaxed state only: LF was higher in men compared to women. Higher body mass index was associated with higher ST at baseline, stress exposure, and in a relaxed state. (Tobacco) smokers showed lower HF under stress exposure than non-smokers.

Since age was the most influencing factor for the measures at baseline and in a relaxed state, we conducted multiple linear correlation analyses with correction for age. In addition, we corrected VLF parameters for respiratory rate (RR interferes with a high frequency of HRV) as well as for sex (significant sex differences). The correlation between openness and VLF was no more significant after multiple correlation analyses (*p* = 0.09).

## Discussion

The main objective of this primary explorative study was to examine electrophysiological correlates of perceived mindfulness as assessed by FMI at baseline, under stress exposure and in a relaxed state. To our knowledge, no study so far has addressed this question.

A deeper understanding of the link between perceived mindfulness and physiological response in MDD might be highly relevant since the physiological reactivity in MDD is altered and MBI aim to improve depressive symptoms.

No correlations between the FMI-total score and the assessed parameters could be observed, contrary to our hypothesis. Interestingly, our finding of a strong inverse correlation between symptom severity and perceived mindfulness (*r* = –0.53) is in line (even exactly the same value) with a meta-analysis comprising 157 distinct samples and 44,075 participants using the Five Facet Mindfulness Questionnaire (*r* = –0.53) (Carpenter et al., 2019).

There are mainly two possible explanations for the presented results according to the literature. First, individuals who score higher in FMI might be better “protected” against emotional dysregulation, ruminations, mind-wandering, self-accusations, and other typical symptoms of MDD (Fountain-Zaragoza et al., 2016; Hsieh et al., 2019; Parmentier et al., 2019; Marion-Jetten et al., 2021; Royuela-Colomer et al., 2021). The other explanation might be that in severe depression, the ability to act mindful is reduced, either because of the impairment of the attentional facet of mindfulness (Teasdale et al., 2000; Elices et al., 2019) or because of the lack of acceptance-related capacities (Eisenlohr-Moul et al., 2016; Kotsou et al., 2018). Nayda and Takarangi (2021) pointed out potential harmful effects of mind-wandering through people’s reduced propensity to be mindful. We assume a combination of both aspects.

In literature, there are first attempts to distinguish the effects of different mindfulness components on the stress level. Lindsay et al. (2018) showed that monitoring plus acceptance training reduced cortisol and systolic blood pressure reactivity compared to monitoring only and control training. In our study, the only correlation between FMI factor “openness” and VLF was no more significant after correction for age, sex, and respiratory rate.

The study has a number of strengths. Participants represent a seriously ill population with a mean BDI score of 30. To our knowledge, findings of the relation between state mindfulness and stress level measured by electrophysiological parameters in different conditions in patients with relevant MDD have not been previously published. Furthermore, we analyzed different possible components of mindfulness, given a solid base for further comparisons with another mindfulness scale, different conditions or other clinical populations.

The following limitations should be mentioned. First, possible confounding effects of respiration (Beda et al., 2014) could not be excluded, since no correction for respiratory rate has been performed in the HRV analysis. However, the respiratory rate was widely within the range of 9–24 breath/min, with exception of some measures mostly in the relaxed state. We conclude that in a relaxed state only, the HRV analysis might be affected by the respiratory bias. Second, the limited power (sample size) can be a potential reason for negative findings. Third, there was no systematic assessment of the individual relaxation strategies.

In summary, despite the heterogeneity of mindfulness definitions, the strong inverse correlation with the symptom severity in depressed patients using FMI in our study was identical with the one in a meta-analysis using different questionnaires (Carpenter et al., 2019), which suggests a robust common conceptual background. The presented study indicates that this inverse correlation between mindfulness and depression severity might not be mediated by stress level, using static as well as dynamic measures (indices of the autonomous nervous reactivity) and self-reported mindfulness.

Our results are in line with previous findings regarding similarities and differences between mindfulness and relaxation responses. Some authors point out, that the effects of mindfulness are to discriminate from stress reduction through relaxation response and do not depend on individuals being in a relaxed state either (citation).

Furthermore, this study underlines the important role of influencing factors in the analysis and interpretation of electrophysiological parameters.

## Conclusion

Although MBI can reduce both stress level and depressive symptoms, the presented results do not support the hypothesis that the bidirectional associated between mindfulness and depressive symptoms in patients with MDD is mediated by ANS reactivity as measured by electrophysiological parameters.

For a better understanding of the physiological effects of mindfulness in MDD, the assessment of autonomous nervous reactivity before and after MBI would be of interest.

## Data availability statement

The original contributions presented in this study are included in the article/Supplementary material, further inquiries can be directed to the corresponding author.

## Ethics statement

The studies involving human participants were reviewed and approved by Ethikkomission Landesärztekammer Baden-Württemberg, Nr. 2018-046. The patients/participants provided their written informed consent to participate in this study.

## Author contributions

JS and AK contributed to conception, study design, and implementation of the study in the clinical setting. JS collected the data, performed the statistical analysis, and wrote the first draft of the manuscript, supervised by AB and UL. AB contributed to the data analysis. UL contributed to the data interpretation. All authors contributed to the manuscript revision, read and approved the submitted version.
